# Overexpressed GATA3 enhances the sensitivity of colorectal cancer cells to oxaliplatin through regulating MiR-29b

**DOI:** 10.1186/s12935-020-01424-3

**Published:** 2020-07-24

**Authors:** Wei Wang, Mei Wang, Jing Xu, Fei Long, Xianbao Zhan

**Affiliations:** 1 Department of Oncology, Changhai Hospital of Shanghai, The Second Military Medical University, 168 Changhai Road, Yangpu District, Shanghai, 200433 China; 2 Department of Oncology, North Ruijin Hospital, Shanghai Jiao Tong University School of Medicine, Shanghai, China

**Keywords:** Colorectal cancer, Oxaliplatin, GATA binding protein 3 (GATA3), miR-29b

## Abstract

**Background:**

GATA binding protein 3 (GATA3) and miR-29b are related to colorectal cancer (CRC). The current study explored the regulatory relationship between GATA3 and miR-29b, and the mechanism of the two in the drug resistance of CRC cells to oxaliplatin.

**Method:**

Apoptosis of CRC cells induced by oxaliplatin at various doses was detected by flow cytometry. CRC cells were separately transfected with overexpression and knockdown of GATA3, miR-29b agomir and antagomir, and treated by oxaliplatin to detect the cell viability and apoptosis by performing Cell Couting Kit-8 (CCK-8) and flow cytometry. The expression levels of GATA3, caspase3 and cleaved caspase3 were determined by Western blot, and the expression of miR-29b was detected by quantitative real-time polymerase chain reaction (qRT-PCR). Animal experiments were performed to examine the changes of transplanted tumors in nude mouse xenograft studies and observed by in vivo imaging. TUNEL staining was performed to detect tumor cell apoptosis.

**Result:**

Both GATA3 and miR-29b agomir inhibited the activity of the CRC cells, promoted apoptosis and Cleaved caspase3 expression, and reduced the resistance of the cells to chemotherapy drug oxaliplatin. Although GATA3 could up-regulate miR-29b expression, the tumor-suppressive effect of GATA3 was partially reversed by miR-29b antagomir. In vivo experiments showed that down-regulating the expression of GATA3 promoted the growth rate and volume of transplanted tumors, while overexpressing GATA3 had no significant effect on tumor growth. TUNEL staining results showed that knocking down or overexpression of GATA3 did not cause significant changes to apoptotic bodies of CRC cells, while oxaliplatin treatment increased the number of apoptotic bodies.

**Conclusion:**

GATA3 inhibits the cell viability of CRC cells, promotes apoptosis, and reduces oxaliplatin resistance of CRC cells through regulating miR-29b.

## Background

Colorectal cancer (CRC) is one of the most common gastrointestinal tumors, and its global incidence has been increasing in recent years [1]. In developed countries, CRC has become the second malignant tumor with the highest incidence just behind lung cancer [2]. Although CRC diagnosis and treatment have been advanced greatly, overall treatment efficacy still needs to be further improved. According to the statistics, the 5-year survival rate after CRC radical surgery is about 50%–60% [3]. Chemotherapy is the main treatment method for patients with advanced CRC and Oxaliplatin (Oxa) is a recommended chemotherapeutic drug to treat patients with high-risk relapse and those with lymph node metastasis [4].

Oxa is a novel platinum anticancer drug [5]. Its platinum atom combines with 1,2-diaminocyclohexane and an oxalic acid group to form a single enantiomeric structure that induces the apoptotic pathway, therefore achieves its tumor cell-killing effect. Oxa is often used for the treatment of metastatic CRC, or in the adjuvant treatment for stage III Dukes C colon cancer after complete resection of the primary tumor [5, 6]. Due to individual differences, many tumor patients still experience cancer recurrence and metastasis after multiple effective chemotherapies, due to tolerance of cancer cells to chemotherapy drugs [7]. Tumor resistance is an important factor affecting the efficacy of tumor chemotherapy, thus, studying the mechanism of tumor cell resistance can help improve chemotherapeutic drugs and treatment effect.

GATA3 is a recently discovered key factor that regulates cell differentiation and cytokine expression [8]. Studies showed that GATA3 not only regulates growth and differentiation of many types of malignant tumors, but also participates in the clinical classification and prognosis of malignant tumors [9–11]. Research revealed that [12] GATA3 is low-expressed in colorectal cancer, and its low expression is associated with high histological malignancy grade, lymph node metastasis, and poor prognosis of CRC. GATA3 is considered to be a tumor suppressor in CRC and involve in miRNAs-mRNA co-regulatory networks of CRC [13]. GATA3 combined with other genes is also identified as a prognostic signature of patients with CRC [14]. GATA3 could activate downstream miRNA-29b and prevent the synthesis of proteins required for tumor metastasis [15]. However, the role of GATA3 in sensitivity of CRC to chemotherapies has been rarely reported.

As an important member of the family of miRNAs, miR-29 has three subunits, namely, miR-29a, miR-29b, and miR-29c, and its coding sequences have high homology in various organisms [16]. MiR-29b-1/miR-29a and miR-29b-2/miR-29c are located on chromosome 7q32.3 and chromosome lq32.2, respectively [17]. Studies reported that miRNA-29b regulates the proliferation of ovarian cancer [18], prostate cancer [19], breast cancer [20] and gastric cancer cells [21], and is related to the occurrence, development and metastasis of these tumors. In CRC, miR-29b targeting MMP-2 is a mechanism, through which hexane extract HAG from American ginseng is able to inhibit colon cancer cell migration [21]. In another study on ulcerative colon cancer, compared with colitis, miRNA-29b expression is significantly increased in tumor tissues, suggesting that it may act as a tumor-promoting gene [22]. Therefore, the role of miR-29b in the development of CRC still remains currently controversial, which requires investigation to the regulation of miR-29b expression and function in CRC and its correlation with tumor biological behaviors.

The interaction between GATA3 and miR-29b had been previously reported in breast cancer cells [23], endothelial cells [24]. The current study compared and explored the molecular expression and interaction relationship between GATA3 and miR-29b, and aimed to determine whether GATA3 regulated the resistance of CRC cells to Oxa through regulating miR-29b. The current findings provide theoretical basis for deciding and optimizing chemotherapy drugs for the treatment of CRC patients.

## Materials and methods

### Ethics statement

All animal experiments were performed in accordance with the Guidelines of the China Council on Animal Care and Use. This study was approved by the Ethics Committee of Shanghai Changhai Hospital (approval number: 201706013ZLX).

### Cell culture

HT-29 (ATCC^®^ HTB-38™) and DLD1 (ATCC^®^ CCL-221™) were purchased from American Type Culture Collection (ATCC), as the two have stable cell functions and are the most commonly used cells in CRC research. HT-29 cells were cultured in DMEM Medium (SH30243.01B, Hyclone, USA) with 10% fetal bovine serum (FBS, 10270-106, Gibco, USA) and 1% double antibody. DLD1 cells were cultured in RPMI-1640 Medium (SH3080901, Hyclone, USA) with 10% FBS and 1% double antibody. The cells were all cultured in a cell incubator (BC-J160S, Boxun, China) at 37 °C with 5% CO_2_.

### Drug treatment of cells

Oxaliplatin (Oxa, 09512) used in the experiment was purchased from Sigma Company (USA). Oxa were added to HT-29 and DLD1 cells at gradient concentrations. HT-29 and DLD1 cell lines were seeded into 6-well plates at a concentration of 5 × 10^5^/ml. After the cells had attached to the wall, the cells were washed twice using PBS, and added with Oxa at gradient concentrations (0 µ mol/L, 1.25 µ mol/L, 5 µ mol/L, 20 µ mol/L, 80 µ mol/L, and 320 µ mol/L). The cells were cultured in a cell incubator for 48 h (h).

### Flow cytometry

CytoFLEX flow cytometer (Backman Coulter, USA) was used to detect changes of cell apoptosis. The cells from different treatment groups were collected after centrifugation (at 250x*g*, 4 °C, 5 min). The supernatants were discarded, and the cells were resuspend in PBS for twice to obtain cell pellet. 500µl Binding Buffer (RVBB-01, Biomiga, USA) was added to the cell pellet to resuspend the cells according to the instruction manual of AnnexinV-FITC/PI Apoptosis Detection Kit (640914, BioLegend, USA). Next, 5µl AnnexinV-FITC was added to the cells, mixed thoroughly and then further mixed with 5µl PI. The mixture was maintained at room temperature away from light for 10 min (min), and the changes of apoptosis were observed and analyzed by flow cytometry. HT-29 cells and DLD1 cells were treated by 50% inhibiting concentration (IC50) of Oxa for subsequent cell processing.

### Construction of GATA3 overexpression (OE) and short hairpin GATA3 RNA (shRNA) recombinant plasmids

To study the effects of GATA3 on CRC cells, we first constructed recombinant plasmids of GATA3-OE and GATA3-shRNA. The GATA3-shRNA sense sequence was 5′-CACCGGACGAGAAAGAGTGCCTCAATCAAGAGTTGAGGCACTCTTTCTCGTCCTTTTTTG-3′ (the underlined part is the target sequence, and “TCAAGAG” is the stem-loop structure). The annealed double-stranded DNA was ligated into the pGpU6/GFP/Neo vector (P05464, miaolingbio, China). The overexpressed GATA3 vector was used to amplify GATA3 by PCR. The sequence of the primers was as follows: forward, 5′-GCCTCTGCTTCATGGATCCC-3′, and reverse, 5′-CTGAGATTCCAGGGGGAGGC-3′. The amplified target gene was ligated with pBR322 vector (N3033L, NEB, USA) in the presence of restriction enzymes Hpa I/EcoRI/Xho I (TAKARA, China) and T4 DNA ligase (20325, TRANS, China). After the plasmid sequencing and identification was completed, plasmids were extracted from the fresh bacterial solution amplified from the cloned colonies using the plasmid large extraction kit (CW2104, Kangwei, China).

### Transfection

Packaging of lentivirus was performed by transient transfection of 293T cells. The day before the experiment, trypisin-EDTA solution (C0201, Beyotime, China) was used to digest and adjust the concentration of 293T cells to 5 × 10^5^/ml. Next, 5 ml of cell suspension was seeded into a 6-well plate. When the cell fusion reached about 80%, 25 µg of the recombinant plasmid (GATA3-OE, GATA3-shRNA and negative control) and 25 µg of PIK were respectively added to the EP tubes. Next 150ul GibicoH_2_0, 50ul CaCl_2_ and 200ul buffer (2 × HBS, pH7.0) were added in sequence. After let stand at room temperature for 25 min, 293T cells and 30µl of chloroquine were added for 24 h, then the collected cell culture supernatant was filtered through a 0.45 pm filter (Millipore, USA) to collect the filtrates. 4 µg/ml polybrene was then added to the filtrates and mixed. CRC cells (4 × 10^5^/well) were inoculated in a 6-well plate 1 day in advance, and the virus supernatants collected every 24 h were added to infect HT-29 and DLD1 cells in succession. GATA3-OE and its negative control were added to HT-29 cells, while GATA3-shRNA and its negative control were added to DLD1 cells. Finally, HT-29 and DLD1 cells stably expressing GATA3-OE and GATA3-shRNA were obtained.

MiR-29b agomir, agomir negative control, miR-29b antagomir and antagomir negative control were all synthesized by RIBOBIO (China). The sequences were as follows: hsa-miR-29b agomir, 5′-UAGCACCAUUUGAAAUCAGUGUU-3′; agomir negative control, 5′-UUCUCCGAACGUGUCACGUTT-3′; hsa-miR-29b antagomir, 5′-AACACUGAUUUCAAAUGGUGCUA-3′; antagomir negative control, 5′-CAGUACUUUUGUGUAGUACAA-3′. HT-29 and DLD1 cells were seeded into 6-well plates (2 × 10^6^/well) containing antibiotic-free medium. When the cell fusion reached 50%–60%, miR-29b agomir and agomir negative control, which had been diluted at 100 nmol/L in PBS, were added to DLD1 cells; while 100 nmol/L miR-29b antagomir and antigomir negative control were added to HT-29 cells [25]. The cells were transfected in an incubator for 24 h at 37 °C with 5% CO_2_.

### Cell Counting Kit-8 (CCK-8) assay

After the transfection, HT-29 and DLD1 were treated by Oxa according to their respective IC50 concentrations, and then incubated at 37 °C with 5% CO_2_ for 48 h. The supernatant was discarded after incubation, and 110 µl of CCK-8 working solution (V_culture medium_: V_CCK-8 stock solution_ = 10: 1, 500T, DOJINDO, Japan) was added to each well to continue the culture for 3 h. The OD value of each well was read using a DG-3022A microplate reader (Nanjing Huadong Electron Tube Factory, China) at 450 nm to calculate cell proliferation rate (cell proliferation rate = OD value of the experimental group/OD value of the control group × 100%).

### Western blot

The cells from different treatment groups were washed twice by PBS [26]. 100 μl of cell lysate (ET111-02, TransGen Biotech, China) was then added to the cells, then vortexed 8 to 10 times on a vortex apparatus, and fully lysed on ice for 5 min. Next, the cells were centrifuged at 1600x*g* and 4 °C for 15 min. The concentration of the obtained protein stock solution (supernatant after centrifugation) was detected by a BCA kit (P0010, Beyotime, China). 100 μg of the proteins were transferred to PVDF membranes by sodium dodecyl sulfate–polyacrylamide gel electrophoresis (SDS–PAGE). The PVDF membranes (0.45uM, IPVH00010, Millipore, USA) were blocked by TBST blocking solution containing 5% skimmed milk powder (66196131T, Yili, China) by centrifuging at a minimum speed for 120 min. 2 ml of blocking solution was added to a 5 ml EP tube, and then added with appropriate amount of primary antibody according to the instructions, and the petri dish was stored at 4 °C overnight. The PVDF membranes were washed by TBST the next day for 10 min for 3 times. Goat anti-rabbit IgG (1: 5000, HA1001, Shanghai Huaan Biological, China) was added to the corresponding bands and further incubated. After incubation for 1 h, the membranes was washed 3 times by TBST. The PVDF membranes were developed by ECL regent (NCI5079, Thermo, USA) for 5 min, and then the X-ray film was pressed, rinsed in developing solution and a fixing solution. Finally the film was developed (XBT-1, Kodak, USA). The primary antibodies and dilution concentrations used in this experiment were as follows: Anti-GATA3 antibody (1: 1000, AF6233, Affinity Biosciences, USA), Anti-β-actin antibody (1: 5000, AF7018, Affinity Biosciences, USA), Anti-Caspase3 + cleaved caspase3 antibody (1: 1000,19677-1-AP, Proteintech, USA). β-actin served as an internal reference.

### Total RNA extraction and quantitative real time-polymerase chain reaction (qRT-PCR)

Each groups of cells were washed them twice using PBS, and the supernatants were discarded. 1 ml of Trizol (15596-018, Invitrogen, USA) was added to the cells, which were then collected into an RNase-free EP tube and centrifuged for 5 min to separate the supernatant (16,000x*g*, 4 °C). 1 ml of supernatant was transferred into a new EP tube. Then, chloroform and isopropanol were added to obtain RNA precipitation. The RNA pellets were washed by ethanol, dried at room temperature for 5 min. After diluting 1 µl of the RNA solution 200 times by DEPC water, the OD260, OD280, and OD260/OD280 values were measured using a UV spectrophotometer (JY02S,Beijing Junyi Dongfang Electrophoresis Equipment Co., Ltd., China), and the purity and concentration of the RNAs were calculated. Next, RNA (0.1µg–5µg) and 1µl oligo (dT) were added to a RNase-free tube, and DEPC water was then added to the tube to a final volume of 12µl. After warming the tube for 5 min to 65 °C, the tube was quickly placed on ice. CDNAs were synthesized by A cDNA synthesis kit (KR201, TIANGEN, China). 4µl 5 × reaction buffer, 2µl 10 mMdNTP mix, 1µl RNase inhibitor and 1µl reverse transcriptase were added to the tube, mixed gently, and incubated with the cDNAs in a water bath 37 °C for 1 h. The tube containing the mixture was then inactivated in a water bath 70 °C for 5 min. The obtained cDNAs were stored in a refrigerator at −80 °C. QRT-PCR reaction system consisted of 12.5 µl SYBR Fast qPCR mix (RR430S, TAKARA, China), 1 µl PCR Forward Primer (10 µmol/L), 1 µl PCR Reverse Primer (10 µmol/L), cDNA 2 µl, and 8.5 µl ddH2O. The reaction conditions were set as follows: at 95 °C for 3 min; at 95 °C for 30 s, at 55 °C for 20 s, at 72 °C for 20 s, for a total of 40 cycles. The final results were expressed by 2^−ΔΔCT^ [27]. The primer sequences used were as follows: has-miRNA-29b-3p forward primer, 5′-TAGCACCATTTGAAATCAGTGTT-3′, and reverse primer 5′-TGGTGTCGTGGAGAGTCG-3′; has-U6 forward primer, 5′-CGCAAGGATGACACGCAAATTC-3′, and reverse primer 5′-TGGTGTCGTGGAGTCG-3’. U6 served as internal reference.

### Animal feeding and weight measurement

Eighteen female SPF-grade BALB/C nude mice (18–20 g, 5 weeks old) were purchased from Shanghai Slark Laboratory Animal Co., Ltd., and the animal certificate number was SCXK (Shanghai) 2017-0005. Nude mice were raised in a laminar flow rack (SPF grade) in the Institute of Brain Functional Genomics of East China Normal University. The feeding, litter disposition, cages and contact equipment used were all autoclaved. The animals were provided with free access to water and food and bred at 23 ± 1 °C in humidity of 55 ± 5%. The experiments started 3 days after all animals adapted to the new environment. The first day of inoculation was recorded as day 1 (D1). During the experiment, the weight changes of all animals were recorded every three days from day 1 to day 26.

### Transplanted tumors by nude mouse xenograft studies

The 18 nude mice were randomly divided into six groups (3/group), namely, Control group, GATA3-OE (OE) group, OE + Oxaliplatin (Oxa) group, scramble group, GATA3 shRNA (shRNA) group, and shRNA + Oxa group. The OE, control, shRNA and scramble groups of mice were injected with corresponding transfected cells. After the cell suspension was thoroughly mixed, 0.2 ml of cells (1 × 10^6^/ml) were added using a 1 ml disposable syringe. After rubbing the left armpit of the mice by alcohol cotton, the cells were injected subcutaneously and inoculated into each group of animals.

### Drug treatment in model animals

Oxa powder was formulated with pure water to a storage concentration of 3 mg/ml, diluted by glucose to a working solution of 0.6 mg/ml and injected into the mice. On the 10th day of the experiment, the animals in the OE + Oxa group and shRNA + Oxa group were injected intraperitoneally with 100 μl Oxa once a week, while the remaining four groups of mice were injected intraperitoneally with the same amount of glucose injection. On the 15th day, all the mice were given 200 µl of Oxaliplatin twice a week. From the 22nd day, the frequency of administration was increased to 3 times a week.

### In vivo imaging

Tumor growth (D10, D15, D19, D24, D26) was observed after cell injection into the mice by in vivo imaging technology [28, 29], and the administration of Oxa was adjusted. Before each imaging, the injection point of the left armpit of the nude mouse was disinfected by 0.1% anal iodine disinfection solution, and the luciferase substrate d-Luciferin (150 mg/kg) was injected into the abdominal cavity using a micro syringe. The nude mice in the experimental group were allowed to move freely for 10 min, allowing d-Luciferin to fully enter the transplanted tumor to react with luciferase via blood circulation. Then the nude mice in the experimental group were placed in an anesthesia box and anesthetized by isoflurane gas. Next, the nude mice were placed in the imaging room, with the tumor in the lateral position and the head inserted into the ventilation tube of the imaging room. Then, the growth of subcutaneous transplanted tumors was observed using an optical imager (IVIS^®^ Lumina III, PerkinElmer, USA).

### Tumor volume measurement and sample collection

The tumor volume was measured using vernier calipers on day 10, 15, 19, 24, and 26. The tumor volumes were calculate according to the volume formula (length × width × width/2), and a line chart was draw. All the animals were sacrificed by intraperitoneal injection of 0.5% sodium pentobarbital solution (P3761-25G, Sigma, USA) by physiological saline, and injected at 50 mg/kg on Day 26. After the extremities muscle reaction disappeared, the growth of the transplanted tumors was recorded using a camera. The mice were in supine position and fully exposed to the transplanted tumor. The transplanted tumor was quickly removed using sterilized surgical instruments, and the shape of the transplanted tumor was recorded using a camera.

### TdT-mediated dUTP Nick-End Labeling (TUNEL) staining experiment [30]

The collected tumors were first fixed by 4% paraformaldehyde (P1110, Solarbio, China), paraffin-embedded and sectioned after gradient dehydration treatment. The sections were treated by Proteinase K working solution (AM2546, Invitrogen, USA) for 30 min at 37 °C, and then rinsed twice in PBS for 5 min each time. TUNEL reaction mixture for each group was prepared as follows: the treatment groups (OE + Oxa and shRNA + Oxa groups) was mixed with 50 µl TdT (EP0162, ThermoFisher, USA) + 450 µl fluorescein-labeled dUTP solution (11420470001, ROCHE, Switzerland); while the negative control group (Control and scramble groups) was only added with 50 µl fluorescein-labeled dUTP solution; the positive control group (OE and shRNA groups) was first added with 100 µl DNase I (11284932001, ROCHE, Switzerland) and reacted at 20 °C for 10 min before adding the TUNEL reaction mixture (11772465001, ROCHE, Switzerland). After TUNEL reaction, the sections were sealed by parafilm and place them in a dark wet box for 1 h at 37 °C. After rinsing the sections in PBS for 5 min for 3 times, the sections were dried and added with 50 µl converter-POD (11772465001, ROCHE, Switzerland). After sealing the sections again using parafilm, the reaction was performed in a dark wet box for 30 min at 37 °C. Next, the sections were rinsed in PBS, added with 50 µl of DAB substrates, and reacted for 10 min at 20 °C. After the reaction, the sections were rinsed 3 times in PBS. Finally, the sections were dehydrated by gradient alcohol, made transparent by xylene and sealed by neutral gum. The changes of apoptosis were observed under a microscope.

### Statistical analysis

The data were analyzed by SPSS 19.0 software (Chicago, IL, USA), and shown as mean ± standard deviation (SD). One-way analysis of variance was used for pairwise comparison. Two-way ANOVA was used for tumor volume data of nude mice analysis. A *p *< 0.05 was considered as a significant difference.

## Result

### Effect of gradient concentration of oxaliplatin (Oxa) on CRC cell apoptosis

**Fig. 1:**
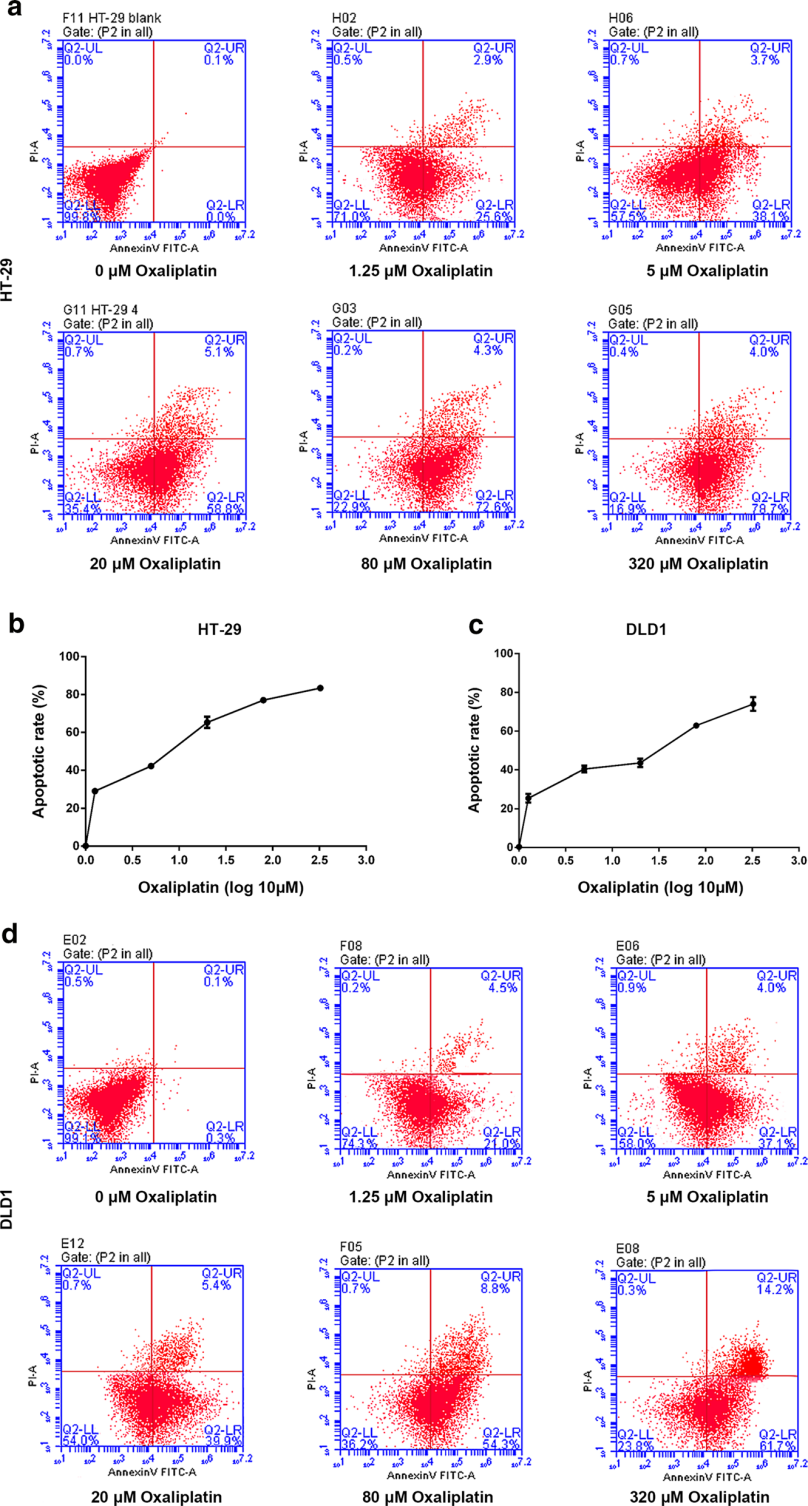
Effects of oxaliplatin (Oxa) at different concentrations on cell apoptosis in each group. **a, b** Flow cytometry was used to examine the effects of different concentrations of Oxa on the apoptosis of HT-29 cells. The X-axis value was the value of log10 for each drug concentration. **c, d** Flow cytometry was used to detect the effects of different concentrations of Oxa on the apoptosis of DLD1 cells. The X-axis value was the value of log10 µM for each drug concentration

To determine the optimal concentration of Oxa treatment, HT-29 and DLD1 cells were exposed to Oxa at a concentration gradient. Figure 1 showed the apoptosis of cells treated by gradient concentrations of Oxa. It can be seen that the apoptosis of HT-29 and DLD1 cells was increased by Oxa in a concentration-dependent manner (Fig. 1a–d). The Oxa IC50 concentration for In HT-29 cells was 11µmol/L (Fig. 1b) and for DLD1 cells was 28 μmol/L (Fig. 1c) for subsequent cell processing.

### GATA3 improves the sensitivity of CRC cells to Oxa chemotherapy

**Fig. 2:**
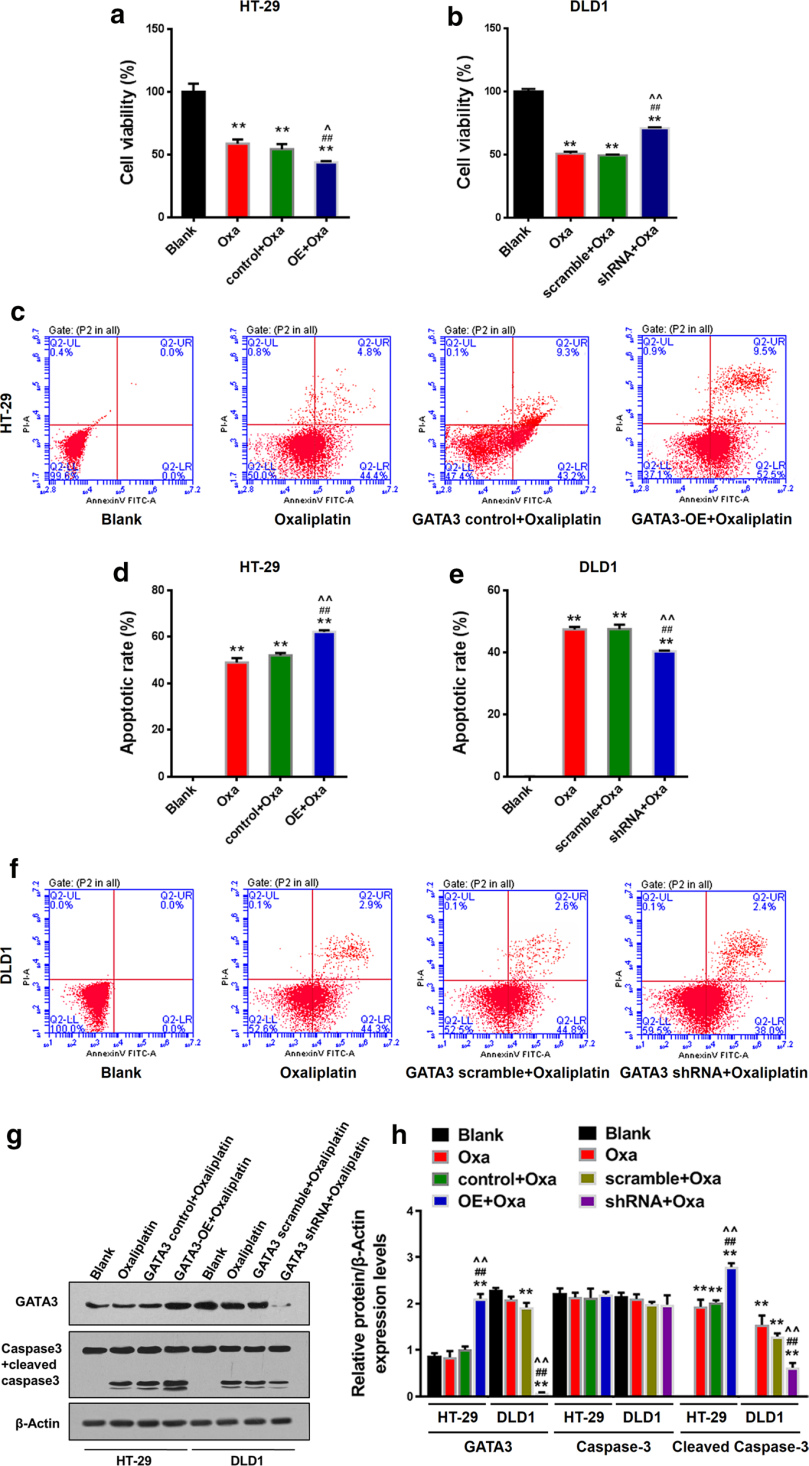
GATA3 improves Oxa chemotherapy sensitivity in CRC. **a, b** HT-29 and DLD1 cell viability in each group. **c–f** Apoptosis and apoptosis rate of HT-29 and DLD1 cells in each group. **g-h** GATA3 and Caspase3 + cleaved caspase3 protein levels in HT-29 and DLD1 cells. The Oxa concentration of HT-29 cells was set to 11 μmol/L; the Oxa concentration of DLD1 cells was set to 28 μmol/L. *OE* Over expression, *Oxa* Oxaliplatin, *shRNA* GATA3 shRNA. Significance of *P* values in **a, d** and **h**: vs. Blank, ^**^p < 0.01; vs. Oxaliplatin, ^##^*p *< 0.01; vs. GATA3 control + Oxa, ^^^^*p *< 0.01. Significance of *P* values in **b, f, h**: vs. Blank, ^**^p < 0.01; vs. Oxaliplatin, ^##^*p *< 0.01; vs. GATA3 scramble + Oxaliplatin, ^^^^*p *< 0.01

Next, we want to know whether GATA3 affect the the sensitivity of CRC cells to Oxa, thus, cell viability and apoptosis were detected. Figure 2a, b showed the cell viability of each group detected by CCK-8. The viability of HT-29 cells in the other three groups treated by Oxa was significantly reduced compared with the Blank group (Fig. 2a, *p *< 0.01). Compared with the Oxa group and the control + Oxa group, the viability of the cells infected with GATA3 overexpression in oxaliplatin-treated group (GATA3-OE) was further reduced (Fig. 2a, *p *< 0.05). Cell viability of DLD1 reduced by Oxa was partially reversed by GATA3-shRNA (shRNA) (Fig. 2b, *p *< 0.01). CCK-8 experimental results showed that GATA3 can significantly inhibit the viability of CRC cells, and knockdown of GATA3 reduced the sensitivity of CRC cells to Oxa.

Figure 2c–f showed the effect of GATA3 on cell apoptosis in each group. Compared with the Blank group, the apoptosis rates of HT-29 cells in the other three groups treated by oxaliplatin were increased (Fig. 2 c, d, *p *< 0.01). Overexpressed GATA3 further increased the apoptosis rate of Oxa-treated cancer cells (Fig. 2c, d, *p *< 0.01). Correspondingly, apoptosis of the Oxa-treated cells transfected with GATA3-shRNA was partially inhibited (Fig. 2e, f, *p *< 0.01). The results revealed that GATA3 promoted apoptosis and reduced the resistance of CRC cells to Oxa.

The protein expressions of GATA3 and Caspase3 + Cleaved caspase3 in each group of cells are shown in Fig. 2g, h. From the figure, GATA3-protein levels of the cells of the GATA3-OE + Oxa group were increased compared with the Blank group, Oxa group, and control + Oxa group (Fig. 2g, h, *p *< 0.01). The expression of Cleaved caspase3 of HT-29 cells (Fig. 2g, h, *p *< 0.01) was up-regulated Oxa and further promoted by overexpressed GATA3 (Fig. 2g, h, *p *< 0.01). GATA3-shRNA reduced the protein expressions of GATA3 and Cleaved caspase3 in DLD1 cells (Fig. 2g, h, *p *< 0.01). Western blot results showed that Oxa treatment significantly increased the expression of Cleaved caspase3 of HT-29 and DLD1 cells, and overexpressed GATA3 can up-regulate GATA3 and Cleaved caspase3 expressions.

### MiRNA-29b improves the sensitivity of CRC cells to Oxa chemotherapy

**Fig. 3:**
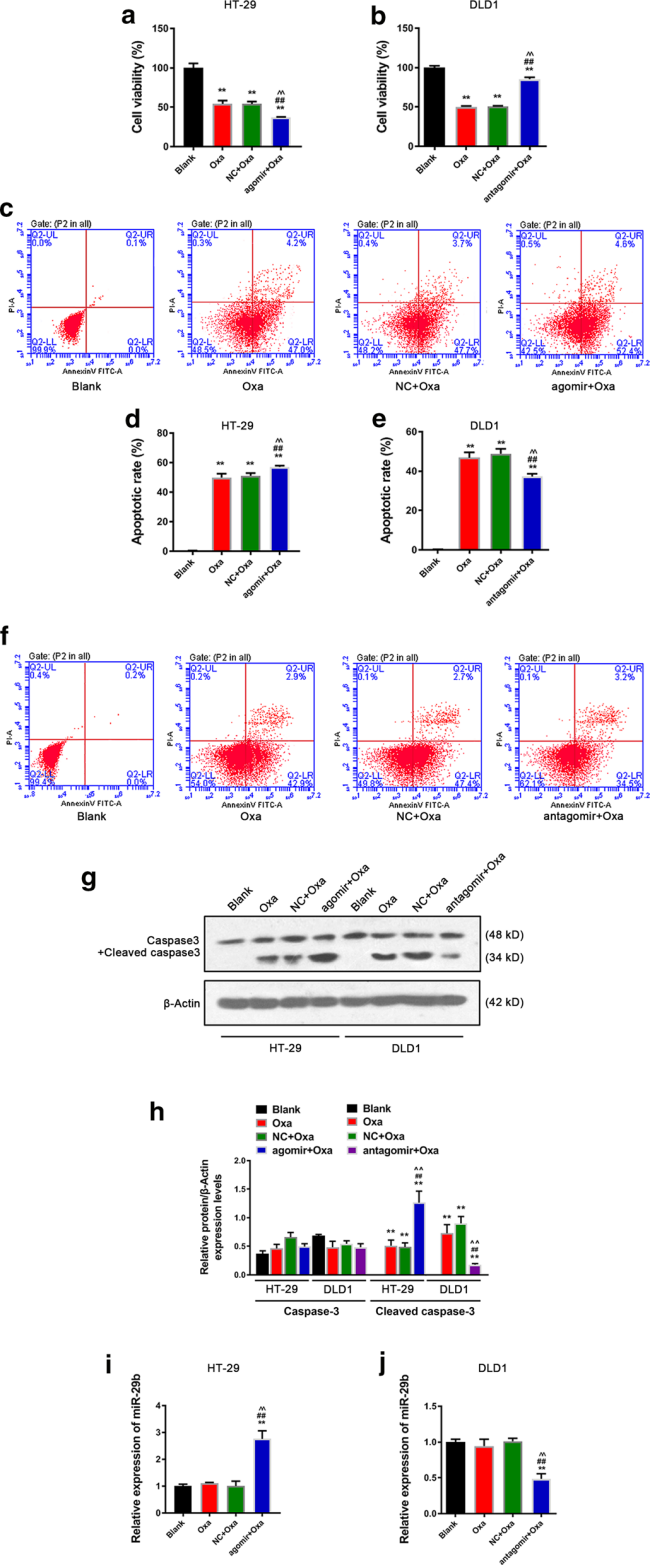
MiR-29b improves CRC sensitivity to Oxa chemotherapy. **a, b** Viabilities of HT-29 and DLD1 cells in each group. **c–f** Apoptosis and apoptosis rate of HT-29 and DLD1 cells in each group. **g–h** Caspase3 + cleaved caspase3 protein levels in HT-29 and DLD1 cells. **i–j** MiR-29b mRNA levels in HT-29 and DLD1 cells. The Oxa concentration of HT-29 cells was set to 11 µmol/L; the Oxa concentration of DLD1 cells was set to 28µmol/L. *Oxa* Oxaliplatin, *NC* negative control, *Agomir* miR-29b agomir, *Antigomir* miR-29b antagomir. Significance of *P* values in **a, d, h, i**: vs. Blank, ^**^p < 0.01; vs. Oxaliplatin, ^##^*p *< 0.01; vs. miR-29b agomir NC + Oxaliplatin, ^^^^*p *< 0.01. Significance of *P* values in **b**, **f, h, i** : vs. Blank, ^**^p < 0.01; vs. Oxaliplatin, ^##^*p *< 0.01; vs. miR-29b antagomir NC + Oxaliplatin, ^^^^*p *< 0.01

To determine the function of miR-29b in sensitivity of CRC cells to Oxa chemotherapy, miR-29b agomir or antagomir was transfected into Oxa treated cells. The viability of HT-29 cells was reduced after Oxa treatment (Fig. 3a, *p *< 0.01) and further inhibited by miR-29b agomir (agomir) (Fig. 3a, *p *< 0.01). Moreover, miR-29b antagomir (antagomir) partially reversed the inhibitory effect of Oxa on the viability of HT-29 cells (Fig. 3b, *p *< 0.01). Obviously, the results revealed that miR-29b can inhibit viability of CRC cells, and overexpressed miR-29b reduced the sensitivity of CRC cells to Oxa.

The effect of miR-29b on the apoptosis of Oxa-treated CRC cells was also explored. We found that miR-29b agomir increased the apoptosis of HT-29 cells treated by Oxa (Fig. 3c, d, *p *< 0.01), but miR-29b antagomir partially reduced the promotion effect of Oxa on the cells (Fig. 3e, f, *p *< 0.01). It showed that miR-29b can promote the apoptosis of CRC cells.

The protein expressions of Caspase3 + Cleaved caspase3 of HT-29 and DLD1 cells were shown in Fig. 3g, h. MiR-29b agomir significantly up-regulated the expression of Cleaved caspase3 of Oxa-treated HT-29 cells (Fig. 3g, h, *p *< 0.01). MiR-29b antagomir greatly reduced the protein level of Cleaved caspase3 of DLD1 cells compared with the other two groups treated by Oxa (Fig. 3g, h, *p *< 0.01). Western blot results showed that miR-29b promoted the expression of Cleaved caspase3 of CRC cells. MiR-29b mRNA levels of HT-29 and DLD1 cells were detected by qRT-PCR, and the data revealed that miR-29b agomir had the biological function of endogenous mature miR-29b and up-regulated the mRNA level of active miR-29b of HT-29 cells (Fig. 3i, *p *< 0.01), while in DLD1 cells, miR-29b antagomir strongly competed with mature miR-29b and down-regulated the mRNA level of active miR-29b of the cells (Fig. 3j, *p *< 0.01).

### GATA3 inhibits the resistance of CRC cells to Oxa through regulating miRNA-29b

**Fig. 4:**
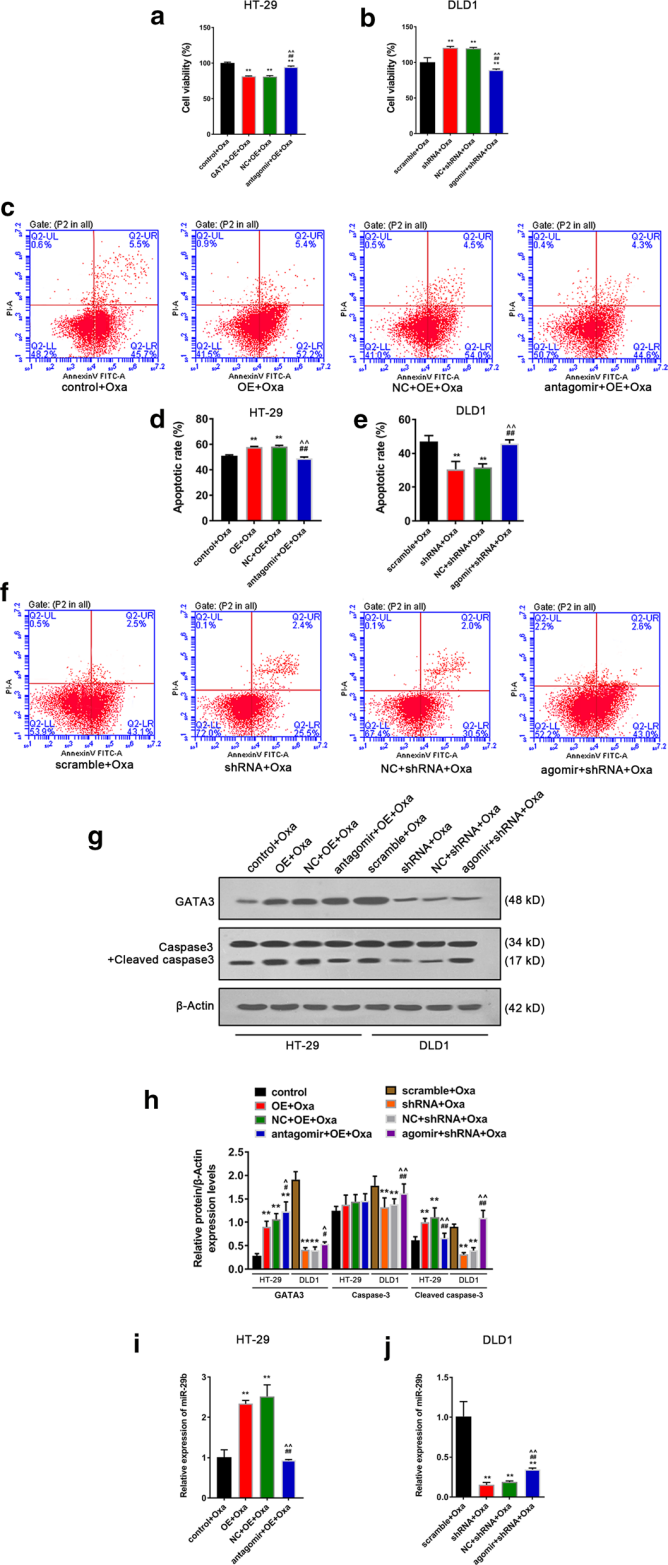
GATA3 inhibits Oxa resistance of CRC cells by miR-29b. **a, b** Viabilities of HT-29 and DLD1 cells in each group. The relative viability of cells normalized to the viability of cells from Scramble + Oxa group. The viability of over 100% meant that the viability was higher than that in Scramble + Oxa group. **c-f** Apoptosis and apoptosis rate of HT-29 and DLD1 cells in each group (**g, h**) GATA3 and Caspase3 + cleaved caspase3 protein levels in HT-29 and DLD1 cells. **i, j** MiR-29b mRNA levels in HT-29 and DLD1 cells. The Oxa concentration of HT-29 cells was set to 11 μmol/L; the Oxa concentration of DLD1 cells was set to 28 μmol/L. *OE* Over expression, *Oxa* Oxaliplatin, *shRNA* GATA3 shRNA, *NC* negative control, *Agomir* miR-29b agomir, *Anagomir* miR-29b antagomir. Significance of *P* values in **a, d, h, i** : vs. GATA3 scramble + Oxaliplatin, ^**^p < 0.01; vs. GATA3 shRNA + Oxaliplatin, ^#^*p *< 0.05, ^##^*p *< 0.01; vs. miR-29b agomir NC + GATA3 shRNA + Oxaliplatin, ^^^*p *< 0.05, ^^^^*p *< 0.01. Significance of *P* values in **a, d, h, i**: vs. GATA3 scramble + Oxaliplatin, ^**^p < 0.01; vs. GATA3 shRNA + Oxaliplatin, ^#^*p *< 0.05, ^##^*p *< 0.01; vs. miR-29b agomir NC + GATA3 shRNA + Oxaliplatin, ^^^*p *< 0.05, ^^^^*p *< 0.01

Next, we were interested in the potential relashionship between GATA3 and miRNA-29b. Thus, a functional rescue assay was cunducted in Oxa stimulated cells after co-transfection of overexpressed GATA3 with miR-29b antagomir or not. The results of CCK-8 demonstrated that compared with GATA3 control + Oxa (control + Oxa) group, cell viabilities of HT-29 cells of the other three groups were significantly reduced (Fig. 4a, *p *< 0.01), while the viability of HT-29 cells of the antagomir + GATA3-OE + Oxa group was significantly increased compared with GATA3-OE + Oxa group and miR-29b antagomir negative control (NC) + GATA3-OE + Oxa treatment group (Fig. 4a, *p *< 0.01). For DLD1 cells, compared with GATA3 scramble (scramble) + Oxa group, the viabilities of the cells of shRNA + Oxa group and agomir negative control (NC) + shRNA + Oxa group were increased (Fig. 4b, *p *< 0.01), however, miR-29b agomir obviously inhibited cell viability (Fig. 4b, *p *< 0.01). The CCK-8 experiment revealed that GATA3 might inhibit CRC cell viability through regulating miR-29b.

It can be seen from Fig. 2c–f that compared with the control + Oxa group, the apoptosis rates of HT-29 cells of the GATA3-OE + Oxa group and the antagomi NC + GATA3-OE + Oxa group were greatly increased (Fig. 4c–d, *p *< 0.01), whereas miR-29b antagomir partially reversed the apoptosis rate of HT-29 cells compared with the antagomir NC + GATA3-OE + Oxa group (Fig. 4c, d, *p *< 0.01). For DLD1 cells, compared with the scramble + Oxa group, the apoptotic rates of the cells of shRNA + Oxa group and agomir NC + shRNA + Oxa group were reduced (Fig. 4e, f, *p *< 0.01), but compared with agomir NC + shRNA + Oxa group, miR-29b agomir obviously increased the apoptotic rates of the cells (Fig. 4e, f, *p *< 0.01). The data indicated that GATA3 enhanced the effect of chemotherapy drugs on promoting CRC cell apoptosis, and the effect could be partially reversed by miR-29b antagomir.

Western blot was performed to detect the effects of GATA3 and miR-29b on the levels of GATA3, Caspase3 + Cleaved caspase3 in each group of cells. The data demonstrated that for HT-29 cells, overexpressed GATA3 increased protein levels of GATA3 (Fig. 4g, h, *p *< 0.01) and Cleaved caspase3 of both GATA3-OE + Oxa group and antagomi NC + GATA3-OE + Oxa group (Fig. 4g, h, *p *< 0.01). However, miR-29b antagomir partially reversed the up-regulated expression of Cleaved caspase3 of DLD1 cells by overexpressed GATA3 (Fig. 4g, h, *p *< 0.01). For DLD1 cells, GATA3 shRNA reduced its GATA3 protein levels (Fig. 4g, h, *p *< 0.01), and its Cleaved caspase3 expression of in the shRNA + Oxa group and agomir NC + shRNA + Oxa group (Fig. 4g, h, *p *< 0.01), however, miR-29b agomir partially reversed Cleaved caspase3 expression of DLD1 cells (Fig. 4g, h, *p *< 0.01). The experimental results revealed that GATA3 further promoted the up-regulation of Cleaved caspase3 by Oxa, and the effect can be partially reversed by miR-29b antagomir.

The miR-29b mRNA levels of HT-29 and DLD1 cells were detected. The PCR results showed that GATA3-OE up-regulated the expression of miR-29b of HT-29 cells (Fig. 4i, j, *p *< 0.01), and miR-29b antagomir competed with miR-29b to down-regulate the active miR-29b level (Fig. 4i, j, *p *< 0.01). For DLD1 cells, GATA3 shRNA down-regulated expression of miR-29b (Fig. 4i, j, *p *< 0.01), miR-29b agomir had the biological function of endogenous miR-29b and up-regulated active miR- 29b level (Fig. 4i, j, *p *< 0.01).

### Evaluation of tumor formation and efficacy of transplanted tumors by nude mouse xenograft studies

**Fig. 5:**
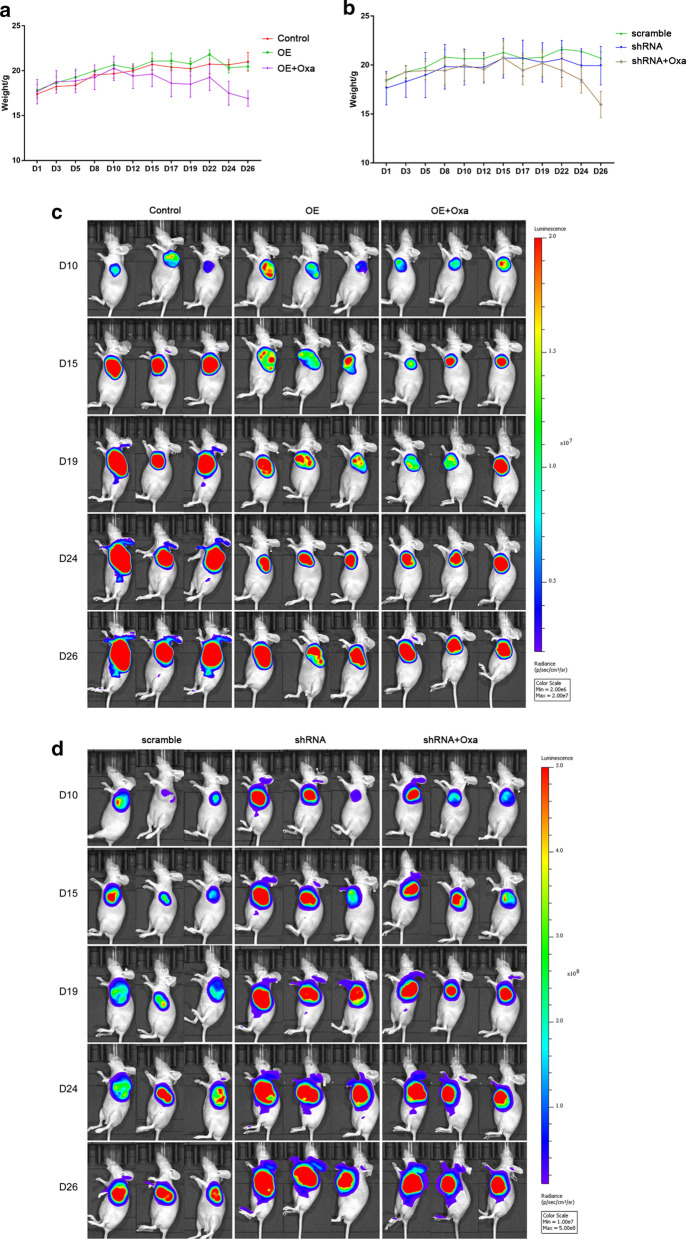
Transplanted tumors by nude mouse xenograft studies were observed. **a, b** Changes in body weight of mice in each group. **c, d***In vivo* imaging results of mice in each group. *OE* Over expression, *Oxa* Oxaliplatin, *shRNA* GATA3 shRNA

**Fig. 6:**
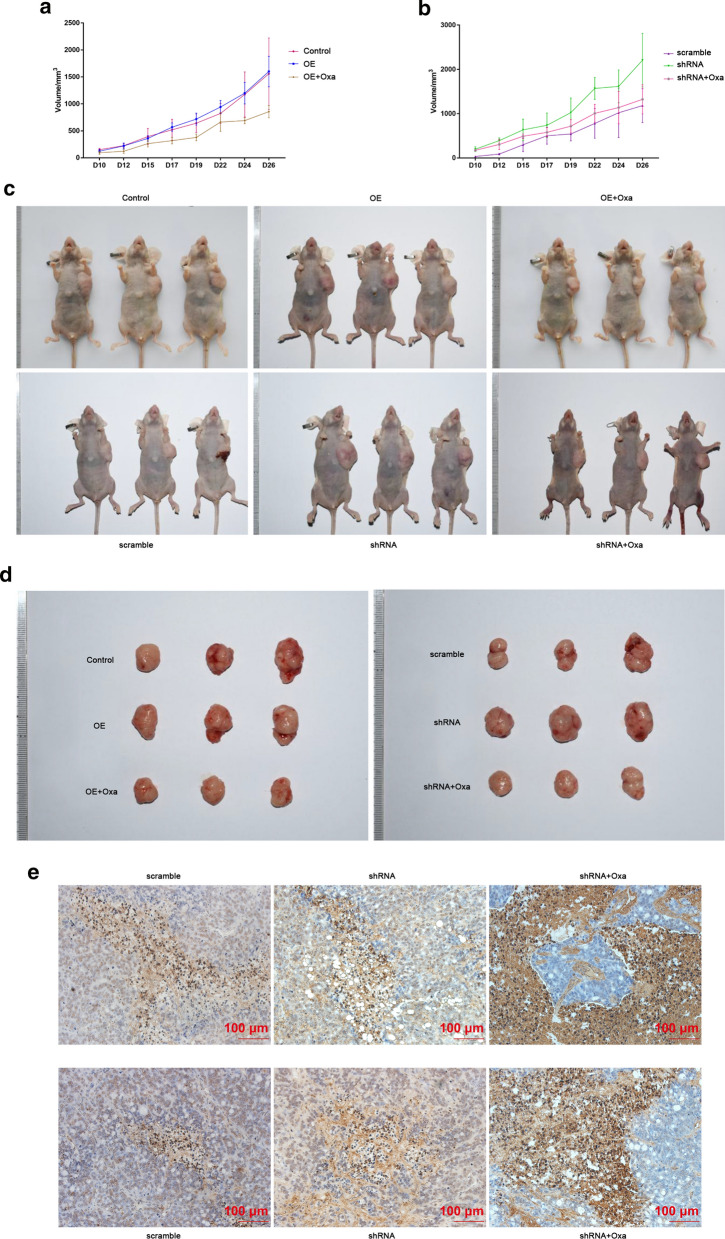
Evaluation of tumor formation and efficacy after transplanted tumors by nude mouse xenograft studies. **a, b** Changes in tumor volume of mice in each group. **c** Changes of transplanted tumors in each group in vivo. **d** Changes in the appearance of transplanted tumors in each group. **e** TdT-mediated dUTP Nick-End Labeling (TUNEL) staining was used to detect apoptotic cells. The magnification was 10 times, and apoptotic cells were marked dark brown

The in vitro assays showed that GATA3 affected the sensitivity of CRC cells to Oxa. However, whether GATA3 had a consistent function in vivo remains unknown. Thus, the xenograft model was conducted with Oxa chemotherapy. From the mouse weight graph, it can be observed that body weight changes of mice with GATA3 overexpression or silence was not obvious, while animals treated by Oxa gradually lost weight (Fig. 5a, b). In vivo imaging results showed that the luciferase activity at the tumor inoculation site increased and gradually spread to the surrounding tissues over time (Fig. 5c, d). As shown in the Fig. 5d, the luciferase activity of the GATA3-shRNA group was enhanced and the area of tumor spread was large. By measuring the tumor volume, we found that GATA3 silencing significantly promoted transplanted CRC tumor growth, while overexpression of GATA3 had no significant effect on tumor growth (Fig. 6a–d), however, Oxa inhibited the tumor-promoting effect of GATA3-shRNA (Fig. 6a–d). The results of TUNEl staining was shown in Fig. 6e, it could be observed that knocking down or overexpression of GATA3 did not cause obvious changes of apoptotic bodies, while apoptosis was visibly increased after Oxa treatment (Fig. 6e). In vitro experiments showed that the resistance CRC cells to Oxa by GATA3 was regulated by the direct inhibition of cell apoptosis.

## Discussion

In order to investigate whether GATA3 was involved in the occurrence of platinum resistance and whether miR-29b was involved in the regulation process, Oxa was used to treat CRC cells with adjuvant and advanced metastasis. The results showed that Oxa significantly increased the protein levels of Cleaved caspase3 of both HT-29 and DLD1 cells. GATA3 obviously inhibited cell viability, promoted apoptosis, and reduced tumor resistance to Oxa, whereas miR-29b partially reversed the role of GATA3 on the two cells, specifically, it up-regulated Cleaved caspase3 protein level, inhibited cell viability, and promoted apoptosis. However, from in vitro experiments, knockdown or overexpression of GATA3 did not significantly affect cancer cell apoptosis, but down-regulation of GATA3 promoted tumor growth and increased Oxa resistance of the CRC cells. The results revealed that GATA3 regulated Oxa resistance through regulating miR-29b rather than by directly inhibiting apoptosis. It was noted that GATA-3 OE + Oxa tretament reduced the viablilty to 80–85% whereas in previous data, the viability dropped to around 60%. We guess the difference between cell state in the two experiments might contributed to the result.

GATA3, which is a specific transcription factor of the Th2 cytokine [31], belongs to the zinc finger structure transcription factor GATA family. GATA1-6 is one of the members that interacts with the common sequence (A/T) GATA (A/G) through higher affinity, and has a DNA-binding motif (zinc finger) [32]. Studies on GATA3 indicated that GATA3 is critical for the growth and differentiation of T cells. GATA3 can promote the differentiation of CD4^+^ T cells towards Th2 during T cell differentiation and inhibit its differentiation toward Th1 [33, 34]. Currently, the role of GATA3 in tumors is focused on malignant tumors of epithelial origin, such as breast cancer, urothelial cancer, prostate cancer, and paraganglioma. Mohammed et al. [35] showed that GATA3 has a high positive rate in aggressive urothelial carcinoma, the positive area is mainly located in the nuclei of malignant cell clusters without false positive, moreover, high expression of GATA3 is positively correlated with large mass. Research by Jiang et al. [36] suggested that the occurrence of prostate cancer is related to the down-regulation of miR-503 through overexpression of miR-503 promoted by GATA3 during epithelial-mesenchymal transition (EMT). In addition, the absence of GATA3 was found to be positively correlated with the highly aggressive nature of breast cancer, for this, research suggested that GATA3 fundtions together with estrogen receptors in producing an inhibitory effects on breast cancer [37, 38]. Chou demonstrated that GATA3 promotes cell differentiation by inducing miR-29b expression, inhibits cancer cell metastasis and changes the tumor microenvironment of breast cancer [23], which is consistent with our findings. The current study observed that GATA3 up-regulated the protein level of Cleaved caspase3 of the CRC cells, inhibited cell viability, promoted apoptosis, and reduced resistance of the cells to Oxa, through regulating miR-29b. Clever Caspase-3 is one of the important apoptotic execution proteins in the Caspase family, and it is a downstream regulatory gene of the Bcl-2 family [39]. It has been reported that GATA3 protein can directly bind to the promoter region of the STAT3 gene and suppress its transcription [40]. Bcl-2 is the downstream target gene of STAT3. Abnormal STAT3 signal can cause the dysregulation of expressions of downstream genes such as Bcl-2 and promote breast cancer progression [41]. Thus, whether the effect of GATA3 on CRC cells was related to STAT3 remained to be further determined.

MiR-29b is widely studied in molecular-targeted therapy of tumors, and it is related to a variety of cancers. The level of miR-29b in primary hepatocellular carcinoma is significantly reduced, and low expression of miR-29b is related to a low differentiation and higher TNM stage of tumor cells, indicating that low-expressed miR-29b is predicative of the degree of tumor differentiation, invasion, and metastasis [42]. MiR-29b acts as a tumor suppressor gene, and it suppresses the expressions of some potential oncogenes through inhibiting methylation and blocking certain pathways [43, 44]. Functional analysis in vitro showed that miR-29b expression is reduced in CRC cell lines and tissue samples, which causes the inhibition of cancer cell proliferation and migration of CRC cells. Proteomic analysis indicated that miR-29b can regulate key biological processes involved in cancer cell metastasis [45]. Therefore, miR-29b is considered as a tumor marker for the prognosis of CRC. In previous studies on tumor chemotherapy drug resistance, miR-29b was found to reverse Oxa resistance of CRC cells by targeting the SIRT1/ROS/JNK pathway [46], moreover, it can also target FOLR1 to inhibit cell growth of colon cancer cells and increases cell sensitivity to Oxa [47]. Our experimental results further complement these previous findings.

At present, malignant tumors seriously threaten human health, and the clinical methods for treating malignant tumors mainly are surgical treatment, chemotherapy and radiotherapy [48]. At present, chemotherapy plays a significant role in cancer treatment, however, drug resistance developed by tumor cells to chemotherapeutic drugs remains a great obstacle, and some patients develop multi-drug resistance of tumors after chemotherapy, which greatly limits treatment efficacy [49]. The current research proved that GATA3 regulated miR-29b to reduce the resistance of Oxa by promoting cell apoptosis and up-regulating the expression of Cleaved caspase3 rather than by directly promoting the apoptosis of CRC cells.

## Conclusion

This study provides a basis for the resistance mechanism of CRC cells to chemotherapeutic drugs, and improved the use of chemotherapeutic drugs. However, there are still some limitations in this study and a lack of related research on the upstream and downstream genes of GATA3, which will be addressed in our future research. Besides, the influence of GATA3/miR-29b axis on colorectal cancer cells to Oxaliplatin should be further confirmed in a xenograft assay.

## Data Availability

The analysed data sets generated during the study are available from the corresponding author on reasonable request.

## Abbreviations

GATA3: GATA binding protein 3
CRC: Colorectal cancer
CCK-8: Cell counting Kit-8
qRT-PCR: Quantitative real-time polymerase chain reaction
OE: Overexpression
shRNA: Short hairpin RNA
SDS–PAGE: Sodium dodecyl sulfate–polyacrylamide gel electrophoresis
TUNEL: TdT-mediated dUTP Nick-End Labeling
Oxa: Oxaliplatin
